# Lipid Raft Size and Lipid Mobility in Non-raft Domains Increase during Aging and Are Exacerbated in APP/PS1 Mice Model of Alzheimer's Disease. Predictions from an Agent-Based Mathematical Model

**DOI:** 10.3389/fphys.2016.00090

**Published:** 2016-03-15

**Authors:** Guido Santos, Mario Díaz, Néstor V. Torres

**Affiliations:** ^1^Systems Biology and Mathematical Modelling Group, Departamento de Bioquímica, Microbiología, Biología Celular y Genética, Instituto de Tecnologías Biomédicas, CIBICAN, Universidad de La LagunaSan Cristóbal de La Laguna, Spain; ^2^Laboratorio de Fisiología y Biofísica de Membranas, Departamento de Biología Animal y Edafología y Geología, Facultad de Ciencias, Unidad Asociada de Investigación ULL-CSIC, Universidad de La LagunaSan Cristóbal de La Laguna, Spain

**Keywords:** Alzheimer's disease, lipid rafts, membrane domains, lipid composition, mathematical modeling, agent based model, DHA, cholesterol

## Abstract

A connection between lipid rafts and Alzheimer's disease has been studied during the last decades. Mathematical modeling approaches have recently been used to correlate the effects of lipid composition changes in the physicochemical properties of raft-like membranes. Here we propose an agent based model to assess the effect of lipid changes in lipid rafts on the evolution and progression of Alzheimer's disease using lipid profile data obtained in an established model of familial Alzheimer's disease. We have observed that lipid raft size and lipid mobility in non-raft domains are two main factors that increase during age and are accelerated in the transgenic Alzheimer's disease mouse model. The consequences of these changes are discussed in the context of neurotoxic amyloid β production. Our agent based model predicts that increasing sterols (mainly cholesterol) and long-chain polyunsaturated fatty acids (LCPUFA) (mainly DHA, docosahexaenoic acid) proportions in the membrane composition might delay the onset and progression of the disease.

## Introduction

Alzheimer's disease (AD) is the most frequent dementia worldwide, affecting more than 40 million people worldwide, with a prevalence of around 10% of the population above 65 years, which doubles every 10 years (Han and Han, 2014). AD is a degenerating irreversible disease that impairs memory and cognitive abilities and, at present, has no cure. AD occurs in two forms: the familial and the sporadic. Although the familial type is known to be associated to several well-defined genetic alterations (Tanzi, 2012; Loy et al., 2014), it does only explain < 1% of AD cases currently diagnosed. Conversely, in the sporadic AD, the most common neurodegenerative form (99% of all cases, Holtzman et al., 2011), the causes are largely unknown, and most evidence indicate that the genome has only a partial contribution on the onset of the disease.

One of the neuropathological hallmarks in AD is the occurrence of senile plaques in neuronal tissue, caused by the accumulation of the neurotoxic amyloid β (Aβ) proteins. Aβ peptides result from the sequential activity of two enzymes, namely β-secretase and γ-secretase (Blennow et al., 2006), which cleavage the transmembrane amyloid precursor protein (APP). According to the amyloidogenic hypothesis of AD, this is a major responsible cause of AD. Aβ peptides become pathological because the 1–40 and 1–42 fragments have a trend to oligomerize and fibrillate, and eventually to precipitate generating neurotoxic aggregates (Rushworth and Hooper, 2010; Claeysen et al., 2012; Hicks et al., 2012) designated as senile plaques. Alternatively, APP protein may be processed in a non-amyloidogenic manner by the α-secretase instead of β-secretase, which release non-amyloidogenic peptides, whose exact function in the normal brain has not been completely elucidated (Claeysen et al., 2012; Postina, 2012).

Furthermore, several recent reports have demonstrated a clear relationship between lipid rafts and amyloidogenic APP processing (reviewed in Rushworth and Hooper, 2010; Vetrivel and Thinakaran, 2010; Hicks et al., 2012). This relationship appears to be tightly linked to the lipid composition and physicochemical properties of lipid rafts (Martín et al., 2010), and is present even at the earliest stages of the disease (Fabelo et al., 2014; Díaz et al., 2015). Lipid rafts are membrane microdomains characterized by a differentiated lipid and protein composition that segregate within the plasma membrane (Brown and London, 2000; Simons and Ehehalt, 2002; Hicks et al., 2012). These chemical features, confer them some particular physicochemical properties including closer lipid packing, rather restricted lateral movement, higher viscosity, and differential thermodynamic properties, compared to the surrounding non-raft regions (Brown and London, 2000; Pike, 2006; Klymchenko and Kreder, 2014). Lipid composition of lipid rafts is the most important biochemical parameter in determining their structure and physicochemical properties (Sonnino and Prinetti, 2013). In particular, the proportion of cholesterol, a key element regarding their maintenance and stability is higher in these domains than in their surroundings. In addition, sphingolipids and saturated fatty acids are notably augmented compared to non-raft membrane domains, these contributing to the higher density, and degree of packing of lipid rafts (Simons and Ehehalt, 2002; Róg and Vattulainen, 2014). Further, long-chain polyunsaturated fatty acids (LCPUFA), including docosahexaenoic acid (DHA), and arachidonic acid (ARA) have been found to be present in lipid raft from nerve cells in the brain of both animal models and humans, yet to much lower amounts than in non-raft domains (Martín et al., 2010; Fabelo et al., 2012), but still they are essential to allow a degree of freedom required for protein-protein and lipid-protein interactions within lipid rafts.

In the context of AD, it is known that the molecules involved in the production of Aβ are segregated inside and outside lipid rafts. In particular, APP is mainly, but not exclusively, located outside the lipid rafts, while the β-secretase and γ-secretase predominantly reside inside (Rushworth and Hooper, 2010; Vetrivel and Thinakaran, 2010; Fabelo et al., 2014). These observations make lipid rafts principal actors in the study of the evolution of AD, since under non-pathological situations, APP is cut outside lipid rafts by the α-secretase and then inside by γ-secretase, while in the pathological condition, APP is sequentially cleavage inside raft domains by β- and γ-secretases (Vetrivel and Thinakaran, 2010; Hicks et al., 2012) This issue have been the subject of many studies aimed to elucidate the role of lipid rafts on the emergence of AD (Rushworth and Hooper, 2010; Zhou et al., 2014), although the precise role of lipid environment in the evolution of AD remains largely misunderstood.

One of the recent findings that pinpoint to lipid rafts as critical elements in the development of AD, has been the demonstration that lipid rafts are subjected to age-dependent changes, which has been coined as “lipid raft aging hypothesis” (Fabelo et al., 2012). Aging is known to be the main risk factor in the development of AD, and the available evidence have demonstrated that the time-course of lipid raft alterations are more pronounced and accelerated in APP/PS1 brains, a familial model of AD (Fabelo et al., 2012). Further, in line with these observations, we have recently demonstrated the presence of altered lipid rafts in human brains, even at the earliest stages of the disease (stages I/II of Braak and Braak). It has also been demonstrated that changes in lipid composition may alter the thermodynamic and physicochemical properties of lipid rafts (Müller et al., 2001; Wood et al., 2002; Diaz et al., 2012), which, on their own, can influence the evolution of AD. Interestingly, in animal models of familial AD, the induced increase in the production of Aβ provokes, in turn, changes in the observed lipid composition (Müller et al., 2001; Wood et al., 2002). Thus, it seems that the system is positively fed back to increase AD neuropathology. In this work we aim to elucidate how changes in lipid composition influence the biophysical properties of lipid rafts and non-raft domains, and how the alterations in lipid rafts might affect the rate of production of Aβ and plaque formation in the evolution of AD. How changes in lipid composition influence?

The experimental analysis of the structure, biophysical properties, dynamic behavior and composition of lipids rafts encompass many difficulties and require different, independent methodological approaches. In this regard the mathematical approach represents a valid and potentially useful alternative to the study of the physicochemical properties and dynamic behavior of lipid rafts and their relation with its composition under normal and pathological conditions. In recent years, several studies have been published using *in silico* approaches (Nicolau et al., 2006, 2007; Burrage et al., 2007; Richardson et al., 2007; Zhang et al., 2008; Herrera and Pantano, 2012; Soula et al., 2012). In some of these contributions, coarse grain models of cell membranes and lipid rafts have been reported (Marrink et al., 2008; Risselada and Marrink, 2008; Perlmutter and Sachs, 2009, 2011; Kucerka et al., 2010; Schäfer and Marrink, 2010; de Joannis et al., 2011; Risselada et al., 2011; Rosetti and Pastorino, 2011, 2012; Baoukina et al., 2012; Fischer et al., 2012; Muddana et al., 2012; Bennett and Tieleman, 2013; Davis et al., 2013; Hakobyan and Heuer, 2013; Marrink and Tieleman, 2013; Barnoud et al., 2014). These studies have allowed unraveling the effects and roles of different types of lipids on the structure of these lipid domains, some of which were confirmed *in vitro* and verified experimentally using artificial membrane vesicles (Barenholz and Thompson, 1980; Finegold, 1993; Maulik and Shipley, 1996). However, at present, there has been no attempt to model the formation of lipid rafts and relate this to the pathogenesis of AD using real nerve cell membranes based on *in vivo* data, thereby using real nerve cell membranes. In this article, we propose a mathematical model to deal with this issue. We ask ourselves here on how the lipid composition of these domains influence the biophysical properties of lipid rafts (measured in terms of size, number, viscosity, and proportion of membrane lipid rafts) during normal aging and also along the evolution of senile plaque formation in animal models of AD. We also inquired on the effect of these biophysical changes on the emergence of AD neuropathology. Finally, based in the mathematical model predictions on the evolution of the physicochemical properties of these domains in AD, we were able to assess how changes in the lipid composition of raft and non-raft microdomains might restore membrane stability and modify AD-like pathogenic processes in an animal model of AD. Our findings lead us to sketch out potential therapies aiming to delay the pathological evolution of AD.

## Materials and methods

### Experimental data

#### Isolation of lipid rafts and non-raft fractions

WT and APP/PS1 mice were sacrificed using carbon dioxide. All experimental manipulations were performed following the procedures authorized by the Ethics Committee for manipulation of laboratory animals at University of La Laguna (Spain). Frontal cortices of four animals from each age and genotype were dissected out and rapidly immersed in liquid nitrogen until membrane domains purification. Lipid raft and non-raft fractions were isolated by ultracentrifugation in sucrose gradients following the protocols described in detail in Diaz et al. (2012) and Fabelo et al. (2012). After differential centrifugation, fractions were collected from top to bottom. The first 2 mL fractions, contained the lipid rafts fractions, while the last fraction of the gradient and the pellet were collected and designated as non-raft fractions. Both fractions were routinely tested for purity in Western blot assays using different lipid raft and non-raft protein markers (i.e., anti-flotillin 1 for lipid rafts and anti-α1 subunit of the Na^+^/K^+^ ATPase for non-raft plasma membrane) following Fabelo et al. (2012).

#### Microviscosity estimates of lipid rafts and non-raft domains

For this purpose we determined the steady-state fluorescence anisotropy of the non-polar 1,6-diphenyl-1,3,5-hexatriene (DPH) following the methodology described in detail in Diaz et al. (2012). Fluorescence polarization spectroscopy was performed using 355 nm excitation filter and 420 nm emission filter in an Appliskan multiplate reader (Thermo Scientific), equipped with appropriate polarizers. Controls containing the fluorophores alone were concurrently examined to correct for light scattering and intrinsic fluorescence. Microviscosity estimates (η) were derived from DPH steady-state anisotropy values using an adaptation of the Perrin equation (Lakowicz, 1999) for rotational depolarization of nonspherical fluorophore as described previously for DPH (de Laat et al., 1977).

#### Lipid raft lipid composition

The lipid composition data (including lipid classes and fatty acids composition) from cortical lipid rafts, non-raft domains, and whole nerve cell membranes both in WT and APP/PS1 animals at the different ages (3, 6, 9, and 14 months) used here, have been recently published by our group (Diaz et al., 2012; Fabelo et al., 2012). Further, based on these data we have also delved into some relevant biophysical and thermodynamic properties of lipid rafts in these same genotypes and ages and reported them in Diaz et al. (2012).

#### Mathematical model

According to our previous findings, the nerve cell membrane of cortical tissue in mouse and human brain has the form of an agent based model, as stated in the approaches previously presented elsewhere (Wurthner et al., 2000; Jicha and Markesbery, 2010). Here, we have considered each element representing a lipid group. Based on previous findings by our group on the involvement and importance of lipids in the development and progression of Alzheimer's disease in humans and transgenic mice models (Martín et al., 2010; Fabelo et al., 2012, 2014) we simplified the membrane lipid matrix and defined five groups of lipids: sterols (cholesterol and sterol esters); n-3 LCPUFA DHA and n-6 LCPUFA (mainly arachidonic acid, ARA); monoenoic fatty acids and saturated fatty acids. Although phospholipids in the plasma membrane are esterified by two fatty acids, we assumed that they move rather independently, which could be a reasonable assumption if there is no much segregation of fatty acids between LCPUFA-containing phospholipid (which in the brain are mainly esterifying phosphatydylethanolamine, phosphatydylserine, and phosphatidylinositol) (Farooqui et al., 2000).

The model represents a fragment of membrane semi-layer (200 × 200 units) with a torus-like topology. In this system, each lipid can interact with four neighbors (up, down, left, and right). There is a given interaction between each pair of neighboring lipids that are calculated based on the non-retarded and additive London-Van der Waals (LVW) attraction (Hamaker, 1937; Israelachvili, 2011). We can assume this type of interactions because forces between lipids within the semilayer occurs between lipid molecules which are very close to each other and because the medium is highly enriched in membrane lipids (see the recent updated review by Nicolson, 2014).

**Figure 1:**
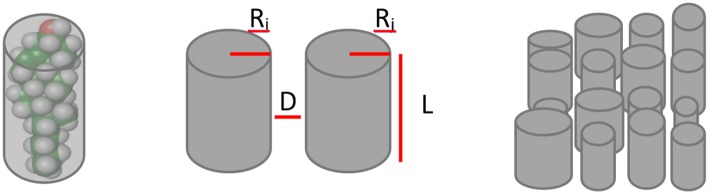
**Lipid groups representation**. Lipid molecules are simplified as the cylinder that best fits their volume and then are parametrized by their radiuses (R_i_, R_j_), lengths (L) and mean distance between them (D). Finally they are disposed in a matrix to simulate the cell membrane.

A key feature in the present agent model is the quantification of the interaction between and amongst lipid groups. In this model, each lipid is represented as the minimum cylinder that can contain it (see Figure 1). This approach has been proven to be useful to predict the behavior of lipids in the cell membrane (Kumar, 1991). Accordingly, the LVW interaction between them is given by Equation 1 (Israelachvili, 2011).

(1) $$\begin{array}{l} {LVW}_{i,j}=\frac{AL}{12\sqrt{2}D^{3∕2}}{\left(\frac{R_{i}R_{j}}{R_{i}+R_{j}}\right)}^{1∕2} \end{array}$$

In this Equation, *A* is the Hamaker constant, with a value between 10^−20^ and 10^−19^ J (in vacuum). *L* represents the length of each lipid. Its magnitude (in Å) corresponds to the length of the lower interacting lipid. *D* is the distance between lipids (in Å) while *R*_*i*_ and *R*_*j*_ represent the radiuses (Å) of the different lipid groups. In the case of saturated fatty acids, its length and width were determined by using Equations (2) and (3) (Israelachvili, 2011):

(2) length=0.154+0.1265n nm

(3) $$\begin{array}{r} \mathrm{volume}=27.4+26.9n\cdot 10^{-3}{\mathrm{nm}}^{3} \end{array}$$

where *n* is the number of carbons; this value being the mean number of carbons in the fatty acids in each lipid group. In the case of unsaturated fatty acids the double bonds increase the volume of the minimum cylinder, as double bonds increase the width of the fatty acids. We have calculated their width based in the number of double bonds and their physicochemical parameters (see Prediction of lipids rafts section). Using Equation 1 and the physicochemical parameters for each lipid, the matrix of interaction forces between pairs of lipids (*LVW*_*i, j*_) was determined. Here, it is worth noting that these physical parameters do not represent the actual dimensions of the molecules, but describe the dimension of the cylinder which best represent the interaction of a lipid group in the context of the cell membrane.

In our model, two adjacent lipids can exchange their positions, the direction of any movement being random. Based in the values of the matrix of interaction (*LVW*_*i, j*_) we were able to assign a probability of movement for each lipid group. For the calculation of this probability we took into account the force keeping each lipid in its current position, this being the sum of all forces acting on the exchanging lipids of the four surrounding molecules. In the case of sphingolipids an additional parameter is required since it is necessary to consider the interdigitation between the two membrane leaflets demonstrated for this lipid class (Maulik et al., 1986) (see later).

The above procedure provides two values of force, which are the corresponding energies that keep each lipid in its current position. In any instance switch movements will occur if both values are lower than a given threshold defined by a uniform probability distribution. This procedure lets more interchanges between pairs of lipids that have lower interacting forces in their current state. Further, each lipid-lipid interaction have a chance to switch, being the lipid to be switched selected randomly.

### Model mobility and lipid rafts

The proposed model was designed to predict the occurrence of lipid rafts, the influence of the membrane lipid composition on this process, the time-course of the changes associated with aging and genotype, and the potential changes in their physicochemical parameters.

We define mobility within the lipid rafts as the quotient number of switching movements observed in each lipid in a 50 times iterations set over 100. Lipids with no movement's restrictions will have 100 switching movements in average (2 movements in each iteration), so values of mobility close to one represent free moving lipids. Accordingly, a mobility value of 1 indicates that the molecule is free to move through, while values lower than 1 indicate the existence of some sort of switching restrictions. This mobility model based measurements can be easily compared with the observed membrane microviscosities measured in nerve cells from animal models, using anisotropy fluorescence spectroscopy (de Laat et al., 1977; Lakowicz, 1999). Repeated model runs showed that a mobility value of 0.36 well describes those regions that can be considered as lipid rafts. Accordingly, regions with mobility values below this threshold will be considered as lipid rafts and values above this value as non-raft regions.

We were able to estimate the model physicochemical parameters (longitude and width) for each group of the model lipids to be used in our model. For this purpose we make use of published data of the membrane lipid composition of neocortex in WT and APP/PS1 mice (Fabelo et al., 2012). Also, based on these data we determined the threshold mobility value to define lipid rafts. For this determination, we used the above indicated initial parameters values. In the case of cholesterol, the physicochemical parameters were estimated using the language of molecular structure modeling *Jmol*. These parameters, which were refined within intervals, were: (1) the dependence of lipid width on the number of double bonds and (2) the width and length of the sterol group. First, an initial search of a 50% above and below the initial value of all parameters let us to restrict the search to intervals where lipid rafts were observed. The best solution predicting the lipid composition of lipid rafts under all conditions found in 2000 solutions was selected.

### Estimation of the number and size of lipid rafts

In order to obtain a measure of the mean size and the approximate number of lipid rafts predicted by the model under each situation. Some properties in the domains must be assumed, these are: (1) Membrane domains are round-shaped, and (2) All lipid raft domains would have the same size.

These assumptions are not very far from the actual situation (see **Figure 3**). Under assumption 1, the area and the perimeter of each domain can be estimated using circle geometry functions, as follows:

(4) $$\begin{array}{r} \text{Area }\left(\text{A}\right)=\text{π}\cdot \text{radiu}{\text{s}}^{2} \end{array}$$

(5) $$\begin{array}{r} \text{Perimeter }\left(\text{P}\right)=2\text{π}\cdot \text{radius} \end{array}$$

On the other hand, under assumption 2, the total perimeter of raft domains as well as their total area can be obtained by counting the number of domains (n), and applying the following equations:

(6) $$\begin{array}{r} \text{Total area}=\text{area }\left(\text{A}\right)\cdot \text{n} \end{array}$$

(7) $$\begin{array}{r} \text{Total perimeter}=\text{perimeter }\left(\text{P}\right)\cdot \text{n} \end{array}$$

Total area and total perimeter can be calculated from data on **Figure 3**. Total area is just the number of spots on the matrix which have a mobility value lower than a previously defined threshold value. The total perimeter was calculated by summing the outcomes of differential function applied to the data of lipid raft identification matrix from left to right and from up to down. The lipid rafts identification matrix was obtained transforming the data of **Figure 3** on a matrix of the same dimension, in which a value of one is assigned when the mobility was under the defined threshold value and zero when it did not.

Once the total perimeter and total area were obtained, Equations (6) and (7) can be replaced on Equations (4) and (5), providing a system of two equations with two unknowns variables (number and radius), which can be easily solved. These number (n) and radius (r) values correspond to the estimations of the number and size of lipid raft membrane domains, respectively.

(8) $$\begin{array}{r} n=\frac{P^{2}}{4\cdot \pi \cdot A} \end{array}$$

(9) $$\begin{array}{r} r=\frac{2\cdot A}{P} \end{array}$$

In these equations, *A* and *P* represent the area and perimeter of the lipid rafts, respectively. Estimates of changes in surface area of non-rafts regions can be obtained by following the changes in radius size of lipid domains (*r*), as we are considering a piece of membrane of constant surface. Increasing values of lipid rafts size (*r*) result in decreasing value of surface area of non-rafts regions.

### The possibility that AD-like pathology can be modified

Based in the model, it was possible to propose a set of changes in the lipid composition of cell membrane microdomains which could eventually displace the parameters obtained for old transgenic APP/PS1 animals toward the values corresponding to those of young Wild-type animals.

The initial pathological condition chosen was that of 9 months old transgenic APP/PS1 mice, which corresponds to a pathological but, not irreversible condition (Diaz et al., 2012; Fabelo et al., 2012). The final, healthy condition, we wanted to move to, was that of 3 months Wild-type mice. To accomplish this, we changed in the mathematical model the composition of each lipid group on the cell membrane domains of the 9 months transgenic mice by 50% in the direction opposing the pathological change in lipid rafts from transgenic mice.

## Results

### Insights into lipid raft composition

**Figure 2:**
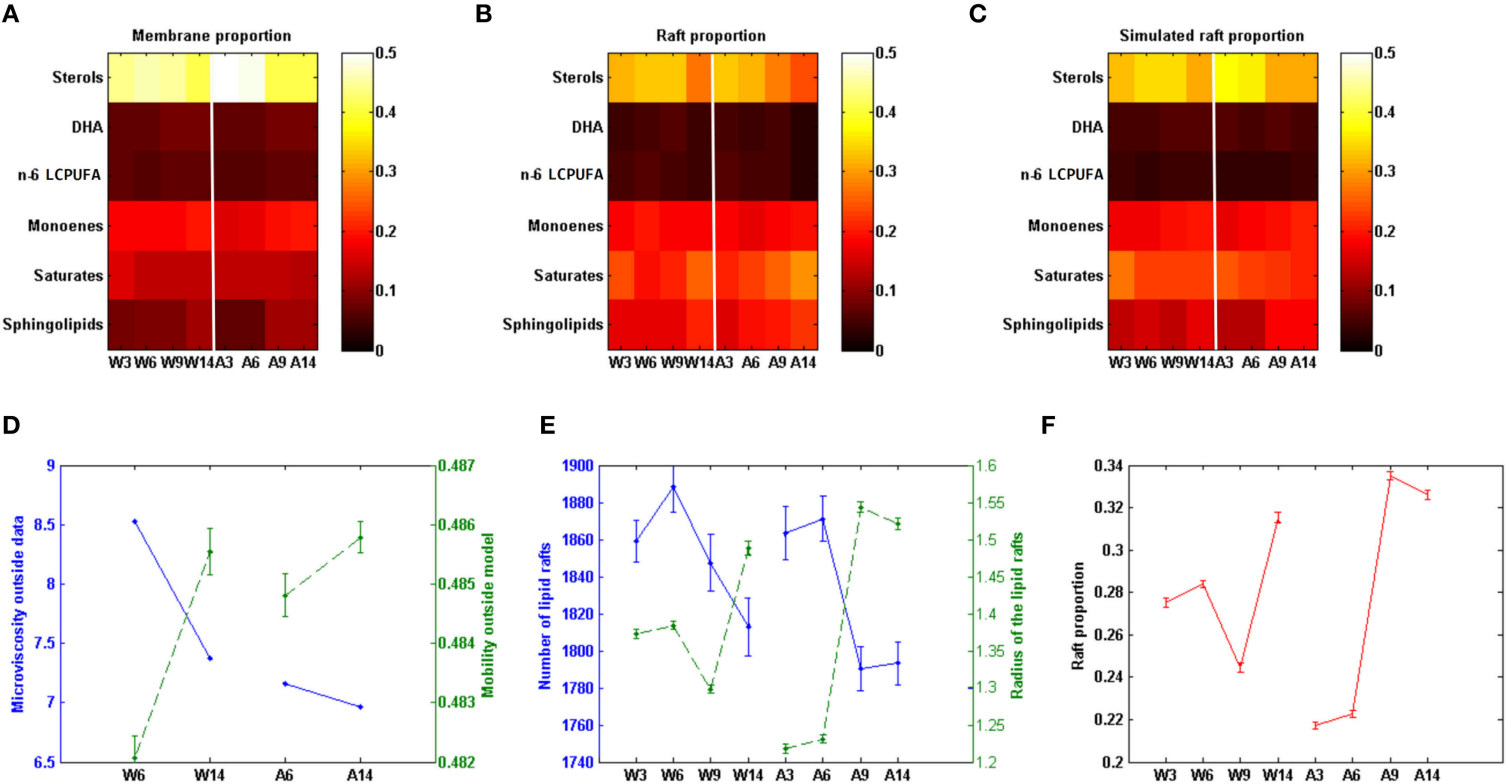
**Comparison of experimental and model predicted membrane features**. **(A)** Lipid groups composition in cell membrane in different conditions (data on Table S1). **(B)** Lipid groups composition in lipid rafts in different conditions (data on Table S2). **(C)** Model predicted lipid rafts composition (data on Table S3). **(D)** Comparison between model based values of mobility (dashed line) and experimental values of microviscosity (continuous line) in the membrane domain (data on Table S4). **(E)** Model predicted number (continuous line) and size (dashed line, as measured by the mean radius) of lipid rafts (data on Table S5). **(F)** Model prediction rafts proportion in membrane. W3; W6; W9; W14: Wild-type mice at 3, 6, 9, and 14 months of age. A3; A6; A9; A14: APP/PS1 transgenic mice of 3, 6, 9, and 14 months of age, respectively. Vertical red lines separate Wild-type and AD induced conditions (data on Table S6). All the experimental data in this figure were taken from Fabelo et al. (2012).

The comparison of some observed and model-predicted membrane features are shown in Figure 2. Figures 2B,C compare the experimentally observed data and the predicted model composition for lipid rafts in WT and APP/PS1 mice (Fabelo et al., 2012), respectively. In addition, it can be seen (Figure 2A) the differences between the observed composition of selected groups of lipids in the whole membrane (Diaz et al., 2012) and the model-predicted composition of the lipid raft (Figure 2C).

**Figure 3:**
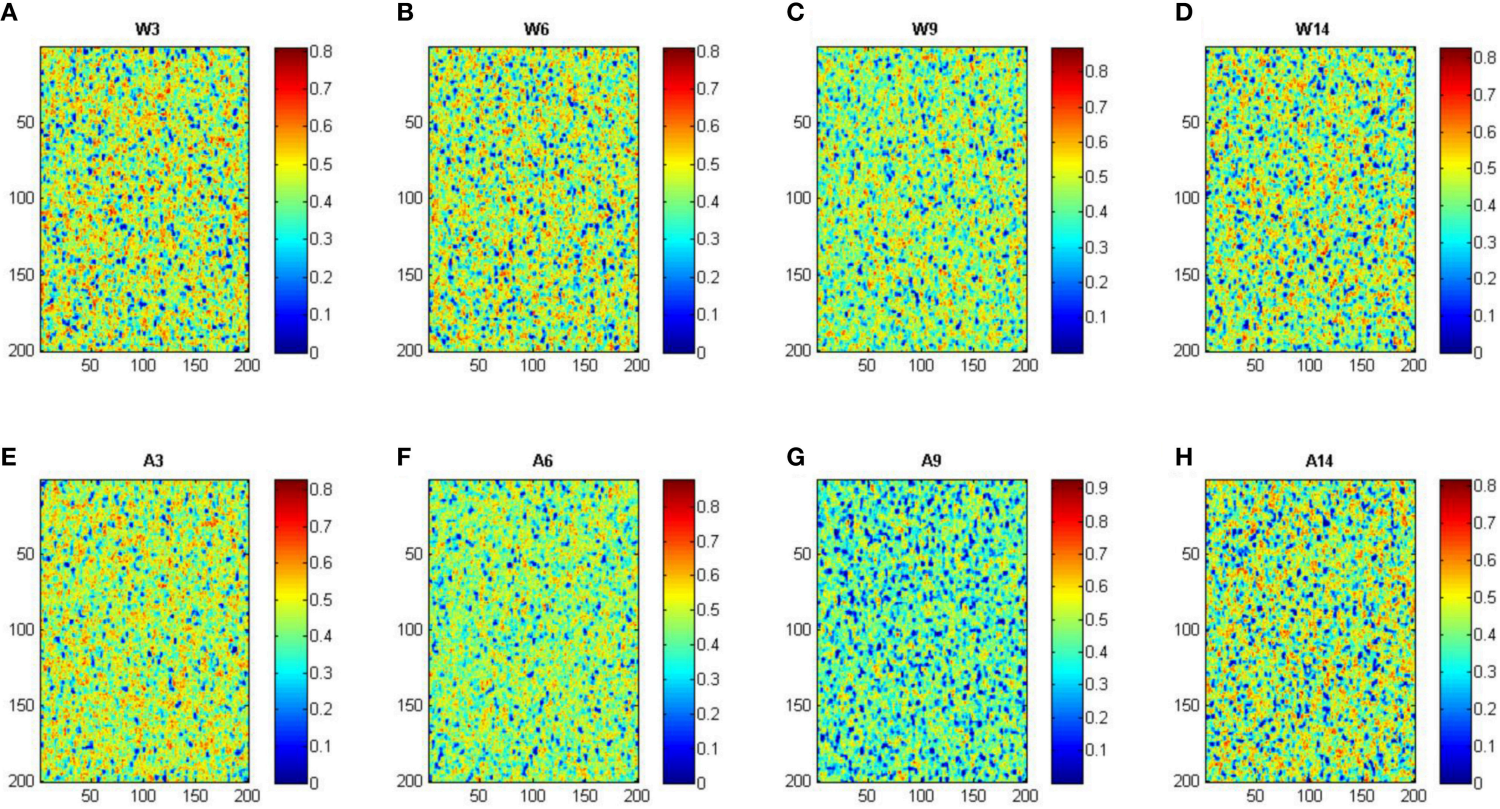
**Simulation of cell membrane lipid composition under different conditions**. W, Wild-type mice; A, APP/PS1 transgenic mice; ages, 3, 6, 9, and 14 months. Color scale indicates mobility. Panels **(A–H)** corresponds to conditions: wild-type 3, 6, 9, and 14 months, APP/PS1 3, 6, 9, and 14 months, respectively.

Based in the model, we have also assessed the predicted membrane lipid structure in WT and APP/PS1 mice under different experimental conditions, including the changes that are found in aging animals. The results are shown in Figure 3.

As it can be seen, the model was able to predict some of the experimental observations. First, there was a good agreement with the observed decrease in the concentration of sterols in lipid rafts with respect to the whole nerve cell membrane. Also, the model reproduced well the observed increase of saturated fatty acids and sphingolipids within the lipid rafts. Finally, the model also showed that the composition of monoenoic fatty acids, DHA, and n-6 LCPUFA on rafts domains and total membrane followed the changes demonstrated experimentally in both Wild-type and APP/PS1 mice (Fabelo et al., 2012).

Sphingolipids are essential and characteristic components of lipid rafts, and partly responsible for the higher degree of lateral packing of lipid within these domains endowing them with a limited lateral mobility (Maulik et al., 1986). Compared with other phospholipids, i.e., phosphatidylcholine, the main structural dissimilarity is chain length in sphingomyelins, where the N-acyl group (usually highly saturated) can be up to 10 carbons longer than the sphingosine (Barenholz and Thompson, 1980). Such a chain disparity give sphingomyelins the unique ability to form both intra- and intermolecular hydrogen bonding and increases the hydrophobic interaction with other membrane components within the membrane plane, and more importantly this property enables sphingomyelins to participate in transbilayer hydrocarbon interdigitation (Slotte, 1999; Sonnino and Prinetti, 2010). In order to characterize and represent this feature we defined the interdigitating parameter and introduced a parameter that decrease its switching between the membrane leaflets, as most sphingomyelin is located in the external leaflets of the membrane. We found that the model predicts that the sphingolipids probability of switching is ~10% lower than for the other lipid groups.

### Lipid shape parameters and Van der waals forces

Table 1 shows the estimated parameters of the set of cylinders that better encase the membrane lipids considered in this study, these including length, width, mean intermolecular distance, and the Hamaker constant.

**Table 1:** **Data based estimation (Diaz et al., 2012) of the cylinders representing the modeled lipid membrane**.

	**Width (radius Å)**	**Length (Å)**
Sterols	5.94	19.99
DHA	6.74	19.01
n-6 LCPUFA	5.84	17.70
Monoenoic fatty acids	5.30	22.59
Saturated fatty acids	6.14	22.44
Sphingolipids	6.18	22.44
Intermolecular distante (Å)	1.5	
Hamaker constant	9.70 × 10^−19^	

Sterols include cholesterol and sterol esters. The Hamaker constant comes from the non-retarded and additive London-Van der Waals (LVW) attraction (Hamaker, 1937; Israelachvili, 2011).

Other relevant parameters for the model are the interaction forces between the same lipid molecules. These interaction values were calculated as indicated by Equation 1 and are shown in Table 2. It can be seen that the strongest interactions occur for saturated fatty acids and sphingolipids, and the weakest for n-6 LCPUFA.

**Table 2:** **Mean force of interaction between lipid groups**.

**Lipid groups**	**Mean forces (N)**
Sterols	1.05 × 10^−18^
DHA	1.04 × 10^−18^
n-6 LCPUFA	9.48 × 10^−19^
Monoenoic fatty acids	1.08 × 10^−18^
Saturated fatty acids	1.12 × 10^−18^
Sphingolipids	1.12 × 10^−18^

Forces are measured in Newtons (N). Sterols include cholesterol and sterol esters.

Also relevant are the interactions amongst different lipid groups (Table 3). In this case, the strongest interactions are observed for sphingolipids and saturated fatty acids. On the other hand, the n-6 LCPUFA group displays the lowest interaction values. Overall, these estimates indicate that lipid raft domains exhibit stronger interaction forces because their particular enrichment in sphingolipids, saturates, and sterols, which is in agreement the restricted lateral mobility and higher compressibility compared to non-raft regions, which, in turn, are depleted of sphingolipids but enriched in polyunsaturated (n-3 and n-6 LCPUFA) whose pair interacting forces are weakest. Therefore, based on the biochemical composition of the model can predict phase segregation and domain formation in nerve cell membranes.

**Table 3:** **Pairs mean force interactions amongst the different lipid groups measured in Newtons (N)**.

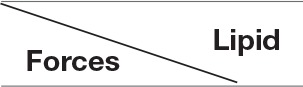	**Sterols**	**DHA**	**n-6 LCPUFA**	**Monoenoic fatty acids**	**Saturates fatty acids**	**Sphingolipids**
Sterols	1.07 × 10^−18^	1.05 × 10^−18^	9.45 × 10^−19^	1.04 × 10^−18^	1.08 × 10^−18^	1.08 × 10^−18^
DHA		1.09 × 10^−18^	9.74 × 10^−19^	1.02 × 10^−18^	1.06 × 10^−18^	1.06 × 10^−18^
n-6 LCPUFA			9.41 × 10^−19^	9.18 × 10^−19^	9.53 × 10^−19^	9.55 × 10^−19^
Monoenoic fatty acids				1.14 × 10^−18^	1.18 × 10^−18^	1.18 × 10^−18^
Saturated fatty acids					1.22 × 10^−18^	1.23 × 10^−18^
Sphingolipids						1.23 × 10^−18^

Sterols include cholesterol and sterol esters.

### Microviscosity quantification in rafts and non-rafts fractions membranes

The experimentally determined values of apparent microviscosity (η_app_) in lipid rafts and non-raft fractions (see Section Materials and Methods) are shown in Table 4. Microviscosity is a membrane environmental property that measures the difficulty for a probe to move in the membrane. We have used the DPH probe as a hydrophobic probe that buries within the membrane core. As it can be seen in Table 4, lipid rafts display higher values of η_app_ compared to non-raft fractions in all groups of animals irrespective of their genotype. However, these values tend to be higher in lipid rafts from APP/PS1 transgenic AD mice. Moreover, paralleling these observations it can be observed that in both genotypes, increased age leads to more viscous domains. It can also be observed that experimental extraction of cholesterol using methyl-β-cyclodextrine (MβCD) brought about a considerable reduction of η_app_ in all fractions, which was more dramatic and severe in lipid rafts, therefore reinforcing the notion that cholesterol (and sterols) and critical components particularly enriched in lipid rafts and largely responsible for their higher viscosity.

**Table 4:** **Microviscosity values (η_app_) of lipid rafts (LR) and non-rafts (NR) domains proved with DPH**.

	**W6**		**A6**		**W14**		**A14**	
	**NR**	**LR**	**NR**	**LR**	**NR**	**LR**	**NR**	**LR**
Microviscosity app (η_app_)	0.847	1.409	0.711	1.937	0.732	1.448	0.691	1.822
Microviscosity app_MβCD_(η_app_)	0.645	0.691	0.602	0.644	0.698	0.748	0.531	0.809
Percent reduction	−23.813	−14.825	−15.329	−42.168	−4.629	−10.206	−23.244	−22.859
Microviscosity ctrl (38°C)	8.525	14.193	7.157	19.503	7.374	14.578	6.962	18.345
Microviscosity MβCD (38°C)	3.416	7.672	3.188	9.722	3.700	6.887	2.811	12.198
APP/WT (6 months old)	83.950	137.412						
APP/WT (14 months old)					94.412	125.834		

MβCD, methyl-β-cyclodextrine.

Based in our mathematical representation we can calculate a closely microviscosity related magnitude, the mobility. Mobility is also a medium property, which measures the facility of movements of molecule in the membrane. It is thus the inverse of the microviscosity. As can be seen in Figure 2D the model predicted mobility values correlates with the experimental microviscosity data. This constitutes *a posteriori* model verification.

### Proportion, number, and size of lipid raft

Based in our model we could determine the ratio of lipid rafts/total membrane and the number and mean size of lipid rafts. Results are show in Figures 2E,D. Figure 2E it can be seen that in Wild-type mice the number of lipid rafts increase until the age of 6 months and then decrease. The observed pattern in APP/PS1 mice is different since the decrease of the number of domains after 6 months is initially sharper than in Wild-type animals and then remains stable. Regarding the mean size of lipid rafts, we find striking differences as well. Thus, in Wild-type mice, the mean radius remained constant until the age of 6 months, but then decreased and lastly the size increased at 14 months. In contrast, in APP/PS1 mice, lipid rafts sizes were smaller than in the Wild-type strain in the youngest animals and then increased significantly after the age of 6 months. Moreover, the evolution of the proportion of lipid rafts on the membrane (Figure 2F) followed the same pattern than the domain size in both Wild-type and APP/PS1 mice. Since the proportion of raft depends on both, the raft size and number, the model thus indicates that the relevant factor here is the size. Interestingly, for both parameters, the time-course of age-related changes in the number and size of lipid rafts, appear to be accelerated in APP/PS1 mice compared to Wild-type animals.

### The possibility that AD-like pathology can be modified

Provided with a reliable model representation we decided to explore what sort of conditions could delay the onset of AD-related lipid alterations, as assessed by the size of lipid rafts being the variable which best correlates with raft proportion (see Figures 2E,F). We explored this in the 9-month APP/PS1 mice. We chose this condition because animal group is older enough to display the pathological changes measured in brain cortex raft membranes (Aso et al., 2012; Fabelo et al., 2012) but at the same time younger enough to allow a reversion of membrane lipids alterations.

**Figure 4:**
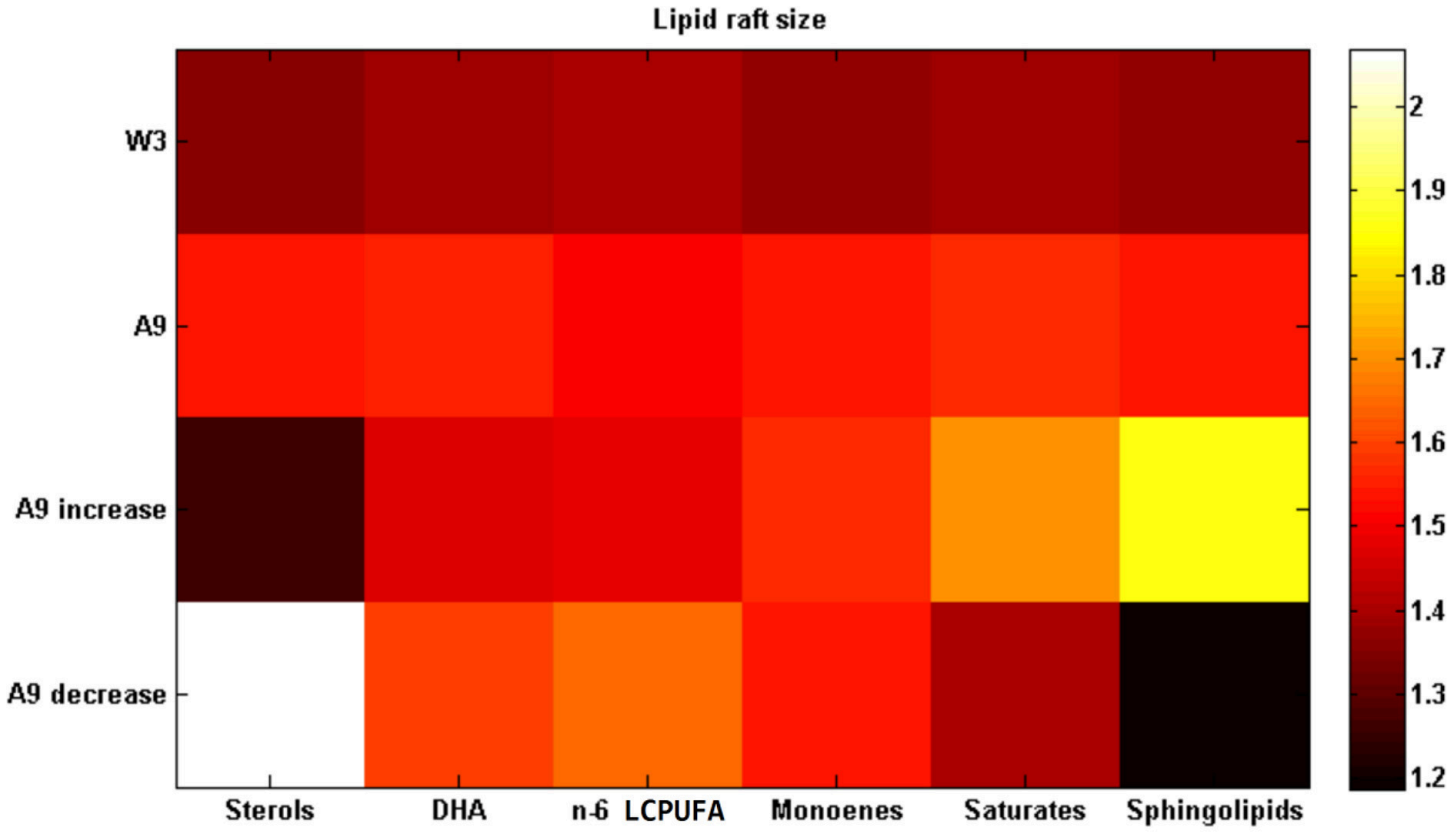
**Changing lipid composition**. Lipid raft size after increasing each lipid proportion by 50% in the 9 months APP/PS1 transgenic mice (third row) or decreasing it (fourth row). First row displays the data from 3 months old Wild-type mice and second row the data from 9 months old APP/PS1 transgenic mice (data on Table S7). All the experimental data in this figure were taken from Fabelo et al. (2012).

Figure 4 shows the results obtained after a 50% change (increase or decrease) in each lipid group and compared with the 3 months old Wild-type mice. It can be seen that there are three lipid groups (sterols, DHA, and n-6 LCPUFA) showing a significant decrease of lipid raft size after an increase of their proportions by comparing first row (3 months Wild-type) and third row (9 months APP/PS1). The other three lipid groups (monoenoic, saturates, and sphingolipids) drive an improvement in raft size when they are reduced (first and fourth rows for WT and APP/PS1 mice, respectively).

## Discussion

In this work we present, the first attempt to quantify the evolution of lipid rafts in Alzheimer's disease by means an agent based mathematical model supported by *in vivo* lipid data composition of nerve cell membranes and their integrated domains, namely lipid rafts and non-raft domains in Wild-type and APP/PS1 transgenic mice at 3, 6, 9, and 14 months. As mentioned before, the original, *in vivo* data used here, derives from our previous work on the detailed analyses of the lipid composition of cortical lipid rafts and whole membrane fractions in these same genotypes and determined at the same ages considered here (Diaz et al., 2012; Fabelo et al., 2012). Moreover, some biophysical and thermodynamic consequences of lipid changes associated with aging have been experimentally determined, particularly in cortical lipid rafts from APP/PS1 animals (Diaz et al., 2012). However, at present there have no modeling attempts to modify lipid compositions and lipid domain formation *in vivo* in order to modify nerve cell membranes in the central nervous system in animal models of AD. The objective of this work was to build up a mathematical model aiming at delving into how molecular alterations in the lipid composition of these domains (raft and non-raft) influence not only their biophysical properties but also their formation and stability. To strength the significance of the predictions, the present agent based mathematical model was calibrated with the real experimental data.

As it has been shown in Figure 2, the model was able to predict the lipid composition of lipid rafts. Lipid rafts microdomains, defined as nanoscopic membrane regions involved in a number signaling processes, with specific lipid composition and differential physicochemical properties compared to their surrounding (non-raft) membrane domains (Brown and London, 2000; Pike, 2006), show mobility values around 30% lower than in the whole membrane or non-raft domains. The model also showed that the presence of sphingolipids provides interdigitation between the two membrane leaflets. The occurrence of this mechanism is responsible of about 10% mobility reduction of sphingolipids, which, in turn, is reinforced by its interaction with saturates and sterols (Barenholz and Thompson, 1980). In addition, since interdigitation influences lateral mobility and compressibility (Maulik et al., 1986), this observation could also explain its stability and higher lipid density within raft domains.

The evaluation of interaction forces between each lipid group and amongst distinct lipid groups (Table 2) show that highly unsaturated lipids (DHA and n-6 LCPUFA groups) exhibit weak interactions between them as well as with sterols, while monoenes, display intermediate interaction force values. At the other end, saturated fatty acids and sphingolipids are the lipid groups showing the strongest interactions. This graduation correlates well with the molecular structures, from the weakest interacting (corresponding to the more twisted molecules, the polyunsaturated fatty acids) to the strongest (the linear; saturated fatty acid).

In addition to this, we have observed that cholesterol strongly interacts with saturated lipids and sphingolipids, which is in agreement with the umbrella effect (Róg and Vattulainen, 2014) (see Table 3). Another model verification comes from the observation that DHA molecules interact more strongly amongst them than with other lipids; an observation in accordance with the role of DHA as dissociating structures in cell domains (Jicha and Markesbery, 2010) which largely contribute to phase separation within the membrane hemilayer (Shaikh et al., 2009; Sonnino and Prinetti, 2010; Georgieva et al., 2015), and likely give rise to the generation of DHA-rich domains bordering (and stabilizing) lipid rafts within nerve cell membranes (Wassall and Stillwell, 2008).

Microviscosity was experimentally determined in lipid rafts and also in non-raft regions surrounding the membrane rafts in Wild-type and AD transgenic mice at different ages (Table 4). The obtained values were found to correlate with the model based mobility values; being these results, at least in part, a sort of experimental model verification.

Furthermore, these observations give new insights regarding the evolution of AD, at least in this transgenic model of familial AD. Thus, we have observed that mobility outside lipid raft domains in AD mice of 6 and 14 months is greater than in Wild-type mice of the same age (Figure 2D). This entails that in the AD conditions lipids and proteins in non-raft regions might move more freely than in lipid rafts, which, in turn, appear to be more viscous, with lipid and protein lateral movements being more restricted in the APP/PS1 mice. In fact, experimental microviscosity values agree with the model predictions.

Based in these observations we propose a mechanism to explain whereby there is an increased production of β-amyloid in AD brain cortex. Since there is a greater molecular mobility outside lipid rafts in these conditions, the entry rate of APP into lipid rafts would be augmented and therefore the rate of amyloidogenic reactions catalyzed within the domain the by β- and γ-secretases would be expected to increase, to the detriment of APP processing in non-raft domains by the α-secretase. This trend increase with aging in WT and APP/PS1 brains, but in AD mice it is of higher magnitude. Since WT mice do not exhibit senile plaques and very low levels of amyloid peptides throughout their lifespan (Aso et al., 2012), there should exist a lipid alteration threshold (probably determined by the level of DHA depletion) above which lipid mobility around lipid rafts would favor the incorporation of APP into lipid rafts. We thus suggest that the increased molecular mobility in non-raft domains is a condition that exacerbates during aging and plays a central role in the observed increase of amyloid β-production in AD patients. In agreement, we have recently demonstrated in human brains that the accumulation of APP and β-secretase in lipid raft is a determinant factor in amyloidogenic APP processing, and that this is an early event in the development of the disease (Fabelo et al., 2014). Also in line with our hypothesis, Das et al. (2013) have demonstrated that convergence of APP and BACE-1 within lipid rafts via an endocytic-dependent pathway triggers the increase of β-secretase cleavage activity, which eventually favors the generation of senile plaques. In addition, the aberrant organization of lipid rafts predicted here in AD brains might be related to the disarrangement of degradative autophagic-lysosomal pathway of β-amyloid peptides observed in AD (Zhou et al., 2014), which, in turn, would facilitate its accumulation.

We have been able also to predict the time-course of age-associated changes in the number, mean size and proportion of lipid raft domains both in Wild-type and APP/PS1 mice (shown in Figures 2E,F). In both genotypes, the number of rafts decreases with aging, but in APP/PS1 mice this occurs earlier and more abruptly, which is in agreement with previous experimental data. However, the good correlation between rafts proportion and raft sizes indicates that the determinant factor to explain the surface increase of rafts is the increase in radius, not its number. Regarding lipid raft sizes and proportion there is an opposite trend with respect to the number of rafts, since the mean radius and proportion increase with age. But, again, the change is sharper in APP/PS1 mice. These results point to the fact that, since we should expect to find a bigger area in the cell membrane as lipid rafts in older individuals, there would exist an increased amount of lipid rafts-associated signaling molecules interacting and likely higher molecular activity in these cases, as suggested by Marin et al. (2013). This situation leads to an unbalanced physiological condition that can be cause and, at the same time, consequence of the aging process. We suggest that homeostatic mechanisms should be operating to control the lipid rafts proportion (in terms of size and number). Perhaps, the reduction of lipid rafts proportion observed in Wild-type mice between 6 and 9 months might be a cellular response to these mechanisms, which appear disrupted in APP/PS1 mice. In the AD mice model the situation becomes somewhat extreme: the increase in rafts proportion occurs earlier and are exacerbated compared to WT. This prediction supports the hypothesis of accelerated “lipid raft aging” that we have previously postulated for this same transgenic mice model of AD (Fabelo et al., 2012). Furthermore, raft proportion in 3 months-old AD mice was predicted to be smaller than in Wild-type animals at the same age. Since in these mice, β-amyloid is produced since the first months of age (Aso et al., 2012), it is suggested that this is an early effect on homeostatic mechanisms controlling the size and proportion of these microdomains. The increase in lipid domains observed later may well reflect the disarrangement of this controlling mechanism. In the same vein as the production of β-amyloid occurs inside lipid rafts, any increase of the raft area during aging would increase the accumulation of all components of amyloidogenic machinery, eventually leading to increased production of β-amyloid. We propose that the increased proportion of lipid rafts is one of the consequences of aging, and that this trend is accelerated in AD patients, which can positively feedback the production of β-amyloid. Interestingly, the prediction that lipid rafts sizes increase prematurely might be extrapolated to human AD patients at early stages of the disease, where not only alterations in the lipid matrix and biophysical properties occur (Díaz et al., 2015), but also the accumulation of APP and γ- and β-secretases as well as their association are increased (Fabelo et al., 2014).

What sort of changes in the lipid matrix could delay or prevent these age-related events? As we stated in the last paragraph, the parameter of domains which best correlates aging and AD evolution is the domain size. This measure was used here as an indicator of the restoring, by comparing for each lipid species the original situation with the situation after modifying one lipid group each time. Taking as reference of the healthy state the 3 month-old Wild-type mice, and the pathological condition to be restored that of the 9 month-old AD mice, we explored different scenarios and found out that the best strategies to partially restore a healthy condition were either, increasing the amount of sterols (mainly cholesterol), DHA, and n-6 LCPUFA or decreasing monoenoic, saturates, and sphingolipids, separately. Figure 4 displays some of these results. It is interesting to note that the healthy effects of the DHA intake on the evolution of AD has been already stablished by a number of epidemiological studies (Jicha and Markesbery, 2010), which constitutes an additional, *a posteriori*, model validation. The other strategy was increasing sterols in lipid rafts. However, the role of cholesterol in the onset and progression of AD remains controversial (Ledesma and Dotti, 2005; Martins et al., 2009; Gamba et al., 2012; Martín et al., 2014) and most studies has not yet been proved to have a positive effect on AD. In fact, most epidemiological and experimental studies on cholesterol have shown a negative effect on AD progression (Martins et al., 2009; Gamba et al., 2012; Maulik et al., 2013). The controversy about the role of cholesterol in AD remains unsolved, however, we have recently postulated a reconciling hypothesis according to which it is the balance between cholesterol and DHA in lipid raft (which affect membrane viscosity of opposite ways), rather than their individual contents, what mostly affect the physicochemical and thermodynamic properties of these domains, eventually impacting the rate of amyloidogenic processing of APP (Díaz et al., 2015).

## Conclusions

The mathematical, agent based model, presented here is able to provide a quantitative assessment of the effect of lipid rafts alterations on some aspects of the progression of AD in relation to the progress of aging. The model was based on previous experimental data of lipid composition and biophysical analyses in whole membrane and lipid domains obtained from Wild-type mice and in a familial model of AD at different ages.

The outcomes indicate that changes in the proportion of lipid rafts (and lipid rafts radius) occur naturally along aging and that this process is accelerated in AD mice, which can positively feedback the production of β-amyloid. We suggest that the mechanisms controlling the domain size are critical in AD evolution. Also, we found increased lipid mobility in non-raft domains associated with aging; this phenomena being even greater in AD. We speculate that both factors, i.e., larger raft domains and higher non-raft lipid mobility would favor amyloidogenic machinery to increase β-amyloid production.

Based on our findings we propose to claim about the importance of sterols and LCPUFA present in the cell membrane domains on the progression of AD and on the potential benefits of these lipid species in delaying the onset of AD.

## Author contributions

GS and NT designed the agent-based model. MD performed experminental work. GS, NT, and MD prepared, discussed, and wrote the manuscript.

### Conflict of interest statement

The authors declare that the research was conducted in the absence of any commercial or financial relationships that could be construed as a potential conflict of interest.

## Abbreviations

η_app_: apparent microviscosity
AD: Alzheimer's disease
APP: amyloid precursor protein
APP/PS1: double transgenic mice model of AD carrying mutated forms of APP and PSEN1
ARA: arachidonic acid
DHA: docosahexaenoic acid
DPH: 1,6-diphenyl-1,3,5-hexatriene.LCPUFA, long-chain polyunsaturated fatty acids
WT: wild-type littermates.
