# Supplementary material for: Lipid Raft Size and Lipid Mobility in Non-raft Domains Increase during Aging and Are Exacerbated in APP/PS1 Mice Model of Alzheimer's Disease. Predictions from an Agent-Based Mathematical Model
Source: Front Physiol. 2016 Mar 15;7:90. doi: 10.3389/fphys.2016.00090 (PMC4791387; doi:10.3389/fphys.2016.00090)
Supplement: Supplementary file 1 [file Table1.pdf]

Supplementary Material

Table S1. Data of Figure 2A.

Membrane proportion data	W3	W6	W9	W14	A3	A6	A9	A14
Sterols	0.44	0.46	0.45	0.41	0.5	0.49	0.41	0.41
DHA	0.07	0.07	0.08	0.08	0.07	0.07	0.08	0.08
n-6 LCPUFA	0.07	0.06	0.07	0.07	0.06	0.06	0.07	0.07
Monoenes	0.18	0.18	0.18	0.2	0.16	0.17	0.19	0.2
Saturates	0.16	0.14	0.14	0.14	0.14	0.14	0.14	0.13
Sphingolipids	0.08	0.09	0.09	0.11	0.07	0.07	0.11	0.11

Table S2. Data of Figure 2B.

Raft proportion data	W3	W6	W9	W14	A3	A6	A9	A14
Sterols	0.32	0.33	0.33	0.27	0.33	0.32	0.28	0.24
DHA	0.04	0.05	0.06	0.04	0.05	0.04	0.05	0.03
n-6 LCPUFA	0.05	0.06	0.05	0.04	0.06	0.05	0.05	0.03
Monoenes	0.18	0.2	0.18	0.18	0.18	0.17	0.18	0.19
Saturates	0.24	0.19	0.21	0.25	0.21	0.23	0.25	0.29
Sphingolipids	0.17	0.17	0.17	0.21	0.16	0.19	0.2	0.22

Table S3. Data of Figure 2C.

Predicted proportion data	W3	W6	W9	W14	A3	A6	A9	A14
Sterols	0.3272	0.3444	0.3515	0.3057	0.3691	0.3660	0.3059	0.3070
DHA	0.0480	0.0516	0.0570	0.0571	0.0547	0.0533	0.0555	0.0534
n-6 LCPUFA	0.0431	0.0371	0.0392	0.0421	0.0381	0.0383	0.0382	0.0432
Monoenes	0.1788	0.1797	0.1917	0.2002	0.1646	0.1815	0.1946	0.2099
Saturates	0.2669	0.2333	0.2275	0.2282	0.2433	0.2341	0.2214	0.2036
Sphingolipids	0.1360	0.1539	0.1331	0.1667	0.1302	0.1268	0.1843	0.1829

Table S4. Data of Figure 2D.

	Microviscosity outside data	Mobility outside model	Mobility outside model (SD)
W6	8.5250	0.4821	0.0004
W14	7.3740	0.4855	0.0004
A6	7.1570	0.4848	0.0004
A14	6.9620	0.4858	0.0003

SD: standard deviation

Table S5. Data of Figure 2E.

	Number of lipd rafts	Number of lipd rafts (SD)	Radius of the lipid rafts	Radius of the lipid rafts (SD)
W3	1,859.0311	11.1610	1.3727	0.0065
W6	1,888.3124	13.6461	1.3841	0.0051
W9	1,847.2337	15.3095	1.2984	0.0062
W14	1,812.7992	15.8212	1.4885	0.0091
A3	1,863.4189	14.1870	1.2182	0.0058
A6	1,871.0945	12.0621	1.2309	0.0057
A9	1,790.2199	11.7272	1.5437	0.0076
A14	1,793.1793	11.3204	1.5215	0.0082

SD: standard deviation

Table S6. Data of Figure 2F.

	Raft proportion	Raft proportion (SD)
W3	0.2751	0.0021
W6	0.2841	0.0018
W9	0.2445	0.0022
W14	0.3153	0.0023
A3	0.2172	0.0015
A6	0.2226	0.0015
A9	0.3350	0.0018
A14	0.3260	0.0023

SD: standard deviation

Table S7. Data of Figure 4.

Lipid raft size	Sterols	DHA	n-6 LCPUFA	Monoenes	Saturates	Sphingolipids
W3	1.3860	1.3763	1.3746	1.3736	1.3890	1.3921
A9	1.5197	1.5376	1.5526	1.5527	1.5466	1.5776
A9 increase	1.2649	1.4564	1.4649	1.5563	1.6963	1.8425
A9 decrease	2.0282	1.6409	1.6078	1.4991	1.3824	1.2015
